# Development of gold nanoparticle-aptamer-based LSPR sensing chips for the rapid detection of *Salmonella typhimurium* in pork meat

**DOI:** 10.1038/s41598-017-10188-2

**Published:** 2017-08-31

**Authors:** Seo Yeong Oh, Nam Su Heo, Shruti Shukla, Hye-Jin Cho, A. T. Ezhil Vilian, Jinwoon Kim, Sang Yup Lee, Young-Kyu Han, Seung Min Yoo, Yun Suk Huh

**Affiliations:** 1 Department of Chemical Engineering, Inha University, 100 Inha-ro, Nam-gu, Incheon, 22212 Republic of Korea; 2 Department of Energy and Materials Engineering, Dongguk University-Seoul, 30 Pildong-ro 1-gil, Seoul, 04620 Republic of Korea; 3 Reliability Assessment Center for Chemical Materials, Korea Research Institute of Chemical Technology (KRICT), 141 Gajeong-ro, Yuseong-gu, Daejeon, 34114 Republic of Korea; 4 Department of Chemical and Biomolecular Engineering (BK21 plus program), KAIST, Daejeon, 34141 Republic of Korea; 5 School of Integrative Engineering, Chung-Ang University, 84 Heukseok-ro, Dongjak-gu, Seoul, 06974 Republic of Korea

## Abstract

A non-labeled, portable plasmonic biosensor-based device was developed to enable the ultra-sensitive and selective detection of *Salmonella typhimurium* in pork meat samples. Specifically, a plasmonic sensor, using the self-assembly of gold nanoparticles (AuNPs) to achieve a regulated diameter of 20 nm for the AuNP monolayers, was used to conduct high-density deposition on a transparent substrate, which produced longitudinal wavelength extinction shifts via a localized surface plasmon resonance (LSPR) signal. The developed aptamers conjugated to the LSPR sensing chips revealed an ultra-sensitive upper limit of detection (LOD) of approximately 10^4^ cfu/mL for *S. typhimurium* in pure culture under the optimal assay conditions, with a total analysis time of 30–35 min. When the LSPR sensing chips were applied on artificially contaminated pork meat samples, *S. typhimurium* in the spiked pork meat samples was also detected at an LOD of 1.0 × 10^4^ cfu/mL. The developed method could detect *S. typhimurium* in spiked pork meat samples without a pre-enrichment step. Additionally, the LSPR sensing chips developed against *S. typhimurium* were not susceptible to any effect of the food matrix or background contaminant microflora. These findings confirmed that the developed gold nanoparticle-aptamer-based LSPR sensing chips could facilitate sensitive detection of *S. typhimurium* in food samples.

## Introduction

The risk posed to human health by foodborne diseases has increased over the last few years^1, 2^, and these diseases cause many human health problems worldwide^3, 4^. Most of these diseases are caused by foodborne pathogenic bacteria, such as *Salmonella typhimurium*, *Escherichia coli O157:H7*, *Staphylococcus aureus*, and *Listeria monocytogenes* ^5^, among which *S. typhimurium* is found commonly in food, meat, and beverages^6, 7^. Indeed, low doses of *Salmonella* cause a wide range of human health problems, including diarrhea, bacterial fever (typhoid), food poisoning, stomach flu, and infectious diseases of the urinary tract, lung, and kidney (septicemia)^8, 9^. The World Health Organization (WHO) reported recently that more than 93.8 million people were affected by foodborne diseases, and approximately 155,000 deaths are caused by these diseases every year^10, 11^. Therefore, it is essential to develop low cost techniques for the sensitive detection and recognition of *S. typhimurium* in mixed populations of foodborne pathogens.

Over the last few decades, numerous approaches have been investigated and used to detect or diagnose *Salmonella* spp., including colony-forming unit (cfu) assays, enzyme-linked immunosorbent assays (ELISAs), and polymerase chain reaction (PCR)-based assays^12, 13^. However, ELISA methods require additional enzyme-supporting antibodies, while PCR-based assays are labor-intensive and time-consuming^14^. Accordingly, several modern alternative techniques have been established to detect or diagnose *S. typhimurium*, such as electrochemical sensors, surface plasmon resonance sensors, fiber-optic biosensors, chemiluminescence, microfluidic devices, impedimetric immunosensors, and conductometric methods^15–17^. Unfortunately, these techniques require well-trained technicians and the identification of the pathogens in large concentrations. In addition, they are time-consuming and expensive, involving complex procedures that do not always provide reasonable results, making them unsuitable for point-of-care needs at resource-constrained, as well as primary care, settings^18^. Therefore, methods for the rapid recognition of pathogens are needed to ensure the quality of all products in the food and agriculture industries.

Recently, plasmonic nanoparticle-based localized surface plasmon resonance (LSPR) biosensors have attracted the attention of many scientists in biotechnological and clinical chemistry because of their high sensitivity, low cost, reliability, reproducibility, and rapid and selective recognition of bacteria^19, 20^. LSPR is a well-known non-labeled detection method, and metal nanostructure-based LSPR sensors have been developed and applied widely in various fields over the last few years^21^. Moreover, the optical LSPR sensor with a metal nanostructured surface is covered with a dielectric environment, which causes incident light matches with the collective oscillation of electrons that stimulate the plasmon peak wavelength^22, 23^. LSPR sensing begins with metal nanostructure surface binding of specific targets to produce plasmon peak shifts^19^. Recently, various types of LSPR sensors have been established and used for the detection of environmentally toxic heavy ions, protein toxins, carbohydrates, caseins, nucleic acids, and biomolecules (biotin-streptavidin), as well as in immune assays^24–27^. However, many of these LSPR biosensors have low reproducibility, and it is difficult to immobilize large areas of plasmonic-active nanoparticles while reducing the detection limit. Although some food and beverage industries have developed new approaches for the simple and rapid recognition of pathogens with excellent sensitivity and improved specificity, to the best of the authors’ knowledge, the combination of gold nanoparticles (AuNPs) and aptamers for use in the LSPR-based detection of *S. typhimurium* has not yet been developed. It has also been reported that LSPR-based biosensors have the advantages of being fast, highly sensitive, label-free, and simple to set up.

In this study, a simple, portable LSPR sensing chip incorporating an aptamer and a large area of AuNPs immobilized uniformly on a transparent substrate was fabricated to monitor *S. typhimurium* contamination. In particular, the aptamer (specific for *S. typhimurium*) was bound to the AuNPs by the dipping method. LSPR sensing chips, which are straightforward, economical, and capable of the rapid identification of *S. typhimurium*, were developed.

In recent years, pork meat has attracted increasing attention as a source of human salmonellosis^28^. In Europe, a recent study estimated that more than 56% of human salmonellosis cases could be attributable to pork meat. Therefore, some European countries have established *Salmonella* surveillance and control programs for pork production^29^. This has increased the demand for the easy monitoring of food and meat samples for the rapid and sensitive detection of foodborne pathogens, with the aim of limiting the number of salmonellosis outbreaks. Therefore, in the present work, the developed LSPR sensing chips were tested for their ability to detect *S. typhimurium* in artificially contaminated pork meat samples. The developed LSPR sensing chip enabled the sensing of *S. typhimurium*, using amine-modified gold nanoparticles and specific aptamers, which might be applicable to environmental monitoring, health care, beverage security, and safety.

## Material and Methods

### Reagents and materials

HAuCl_4_ (≥99.9%) and (3-aminopropyl)-triethoxysilane (APTES; ≥9 8.0%) were purchased from Sigma-Aldrich (USA). Trisodium citrate dehydrate was obtained from Kanto Chemical Co., Inc. (Japan) and methyl alcohol (99.5%) was acquired from Samchun Pure Chemical Co., Ltd. (Korea). Glass substrates, and all other chemicals were of analytical grade and used as received. Phosphate-buffered saline (PBS, pH 7.4) was prepared using 0.01 M Na_2_HPO_4_ and 0.01 M NaH_2_PO_4_. Ultraviolet/Visual (UV/Vis) spectrophotometry was conducted using a V-770 spectrophotometer (Jasco International Co., Ltd, Japan). Field emission scanning electron microscopy (SEM, S-4300, HITACHI, Japan) images were also taken.

### Bacterial strains and culturing

In this study, *S. typhimurium* strain KCTC 2421, which was obtained from the Korea Collection for Type Cultures (Korea), was used in the development of the gold nanoparticle-aptamer-based LSPR sensing chips. *Lactobacillus acidophilus* (KCTC 3164) and *Pseudomonas aeruginosa* (ATCC 15692) were also used to analyze the effect of background microflora on the detection of *S. typhimurium*. All strains used in this study were cultured in Luria-Bertani (LB) medium for 18–20 h (or overnight) at 37 °C on a shaking incubator (200 rpm).

An overnight grown culture of *S. typhimurium*, as a stock concentration, was quantified by a nine-fold dilution in PBS and spreading the dilution onto LB agar plates, followed by incubation at 37 °C overnight. Finally, the individual *S. typhimurium* colonies were counted, and the concentration of the overnight culture was calculated as 10^9^ cfu/mL. The appropriate concentrations in the experiments were obtained by diluting the culture in PBS.

### Selection of aptamers

The *Salmonella typhimurium* (*Sal*), *Lactobacillus acidophilus* (*Lac*), and *Pseudomonas aeruginosa* (*Pse*) aptamers were purchased from the Korea Testing & Research Institute, Korea. In the present study, we successfully developed an aptamer-based LSPR sensing strategy for the detection of foodborne pathogenic bacterial strain *S. typhimurium*. Further, in order to confirm the applicability of the developed strategy for the detection of other pathogenic strains, we chosen *Lactobacillus* (higher cell numbers which may decompose food during fermentation by producing higher amounts of acids) and *Pseudomonas* (frequently reported contaminant bacterial pathogen in foods and drinking water) bacterial strains as target pathogens, and their specific aptamers were designed. The sequences of the bacterial species-specific aptamers are shown in Table S1.

### Preparation of gold nanoparticles (AuNPs)

The AuNPs were synthesized as described previously^30^. Briefly, 150 mL of freshly prepared boiling trisodium citrate (2.2 mM) was added dropwise to 1 mL of a HAuCl_4_ solution (25 mM). The resulting particles were coated with negatively charged citrate ions. The color of the solution changed to wine red, suggesting the fabrication of monodisperse spherical particles. The colloidal HAuCl_4_ solution was then cooled to room temperature without stirring and preserved in the dark at 4 °C. In addition, the morphology and size of the gold nanoparticles were analyzed by transmission electron microscopy (TEM).

### Preparation of LSPR chip surface

The LSPR sensing chip was prepared on a glass substrate of 5 cm × 0.8 cm (length × width). The glass substrate was immersed in methanol and subjected to ultrasonic treatment for 15 min to remove impurities. The substrate was then washed three times with distilled water and methanol, after which the substrate was coated with 0.5% APTES, as an amine group linker, at 50 °C for 1 h. The glass substrate was then washed five or six times with distilled water to remove the loosely bound APTES, after which it was immersed into an AuNPs solution for 8–9 h. The color change of the amino functionalized glass substrate surface from colorless to burgundy was monitored, which indicated the adhesion of AuNPs onto the amino functionalized glass substrate surface.

### Optimization of the aptamer concentration and incubation period for the AuNPs- functionalized LSPR chip surface

To functionalize the chip surface of the developed LSPR, the concentration of the specific aptamers needed to be optimized. To fix the thrombin binding aptamers (80 μM stock concentration) on the AuNP-functionalized glass substrate surface, 400 µL of various concentrations of aptamers (0, 0.01, 0.1, 1, 10, and 20 μM) were diluted with PBS buffer and the resulting LSPR chips were then immobilized with the aptamers at room temperature. The chips were incubated for various times (5, 10, 15, 20, 25, 30, and 60 min) to immobilize the aptamers. Based on the LSPR peak intensity, the appropriate aptamer concentration and incubation period were selected. Subsequently, the aptamer-immobilized sensing chips were rinsed with distilled water. Water contains fewer hydrocarbons and contaminants that could influence the sensing process; therefore, all buffer solutions were made using ultrapure water (Millipore). After selection of the proper incubation period for aptamer conjugation, the sensing chip was tested for its UV/Vis spectra. The control used was the same AuNP-functionalized LSPR chip surface without conjugated aptamers. The spectra were collected using the same method described above.

### Assay format

The format of the newly developed gold nanoparticle-aptamer-based LSPR sensing chip assay consisted of fresh cultures of *S. typhimurium* and a glass slide fabricated with specific aptamer-conjugated gold nanoparticles. The assay was performed at room temperature by dipping the prepared sensing chip into the *S. typhimurium* test culture (500 µL) followed by incubation for 30 min. Subsequently, The LSPR peak intensity was then measured using a UV/Vis spectrophotometer. The complete detection process took approximately 30–35 min.

### Sensitivity test of the gold nanoparticle-aptamer based LSPR sensing chip

To analyze the sensitivity of the developed gold nanoparticle-aptamer-based LSPR sensing chip, a pure culture of *S. typhimurium* was diluted serially (10^3^, 10^4^, 10^5^, 10^6^, 10^7^, 10^8^, and 10^9^ cfu/mL) and the prepared sensing LSPR chip was soaked in each bacterium concentration separately for 30 min to achieve the proper binding of gold nanoparticle-aptamer and the test bacterium. The LSPR peak intensity was then measured using a UV/Vis spectrophotometer. The detection sensitivity of the developed assay was determined based on the LSPR peak intensities. All experiments were repeated at least three times.

### Specific detection of *S. typhimurium* using gold nanoparticle-aptamer based LSPR sensing chip

To examine the specificity of the developed gold nanoparticle-aptamer-based LSPR sensing chip, as a pre-test, overnight grown pure cultures of *L. acidophilus* and *P. aeruginosa*, were diluted serially to final concentrations of 10^0^ to 10^8^ cfu/mL and then used to check the specificity of the developed assay format by analyzing the LSPR peak intensities. Further, to perform genus specific cross-reactivity, test other strains of *Salmonella* species at 10^8^ cfu/mL were also been tested. The experiments were performed in triplicate.

### Food trials

#### Artificial inoculation of *S. typhimurium* into pork meat samples

To verify the practical application of the developed gold nanoparticle-aptamer-based LSPR sensing chip, a stock bacterial suspension of *S. typhimurium* was cultured in LB and incubated at 37 °C for 24 h. The cultures were then diluted serially to prepare the desired concentrations of *S*. *typhimurium* (10^2^, 10^3^, 10^4^, 10^5^, 10^6^, and 10^7^ cfu/mL), which were used in further experiments. For each parameter, 200 g of pork meat was added aseptically to a beaker, mixed with 1000 mL of distilled water, and spiked separately with 1 mL of a culture of *S. typhimurium* at various concentrations, followed by mixing^36^.

#### Detection of *S. typhimurium* in pork meat samples by the aptamer-based LSPR chip

The gold nanoparticle-aptamer-based LSPR sensing chips were used to detect the *S*. *typhimurium*-spiked pork meat samples. A 400-µL sample of each artificially contaminated pork sample from the beaker was dispensed and the contaminated pork samples were then tested using the developed gold nanoparticle-aptamer-based LSPR sensing chip. *S. typhimurium* was detected by analyzing the LSPR peak intensity in a UV/Vis spectrophotometer. Non-spiked pork samples were used as a negative control. All experiments were performed in triplicate to maintain the reliability and reproducibility of the work.

### Statistical analysis

The detection limit was calculated as the mean value of the LSPR peak intensity at zero concentration with three standard deviations^31^. All the results are presented as the average ± mean standard deviation of triplicates.

## Results and Discussion

### Preparation of gold nanoparticles (AuNPs)

The synthesis of nanoparticles with the desired size/shape is very important, particularly in the emerging field of nanotechnology^32^. The AuNPs were synthesized with a controlled size (using various approaches, such as reduced precursors, and varying the Au (III) ion to stabilize it) that was useful for sensitive detection, according to a previously reported method^28^. As shown in Figure S1, the TEM image and UV/Vis spectrum showed that the size of the prepared AuNPs was 20 nm. The surface plasmon absorption spectrum of the AuNPs showed a maximum adsorption peak at 523 nm, confirming the proper production procedure and the size of the gold nanoparticles. Furthermore, the nature of the particle stabilizer, solvent system, and reaction conditions (e.g. pH and temperature) play crucial roles in determining the final size of the AuNPs. In addition, the size of the gold nanoparticles has potential effects on the detection of analytes, as smaller nanoparticles provided a better detection range (data not shown).

### Preparation of LSPR surface sensing chip

**Figure 1:**
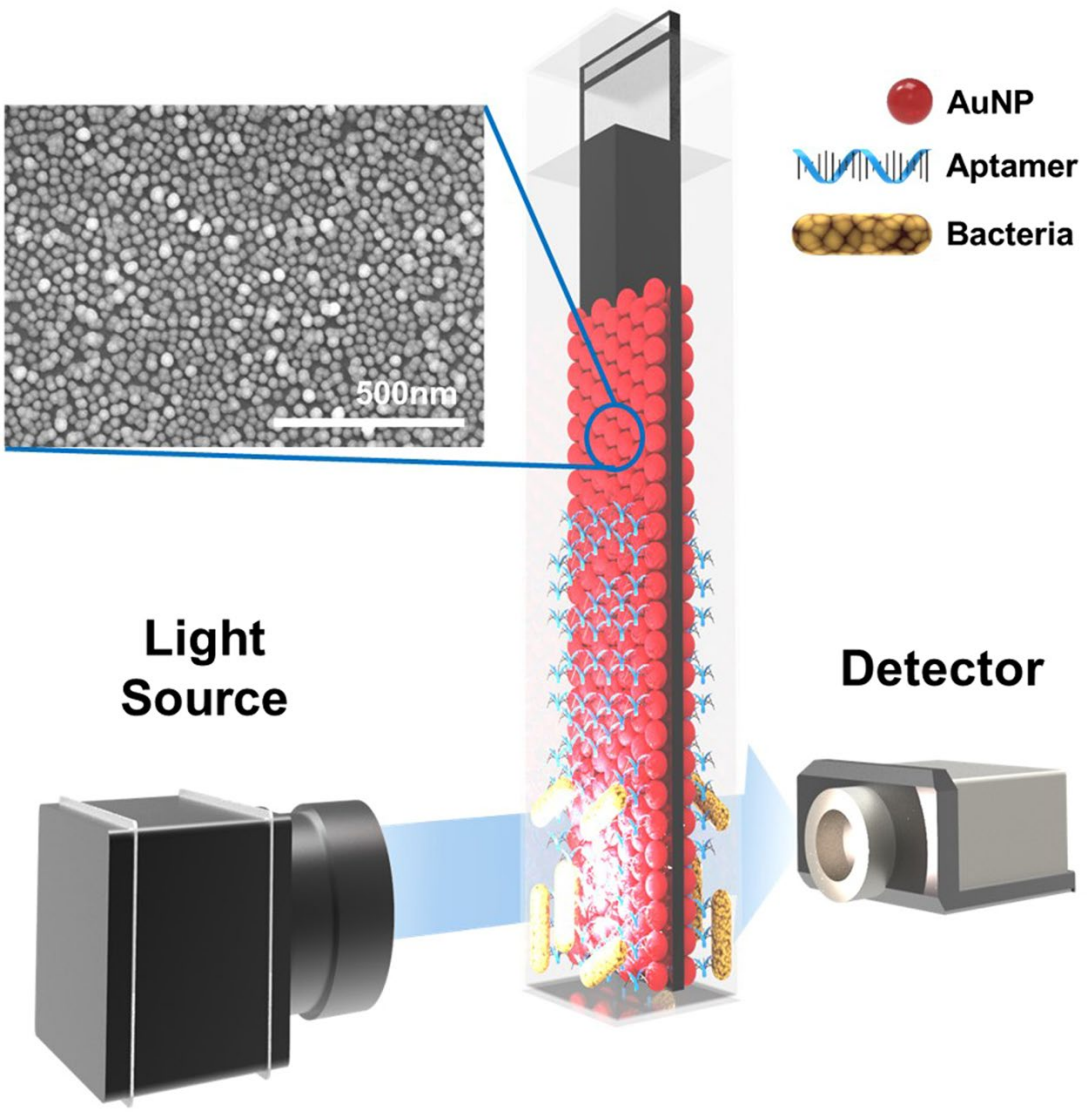
Schematic representation of the detection of bacteria using the localized surface plasmon resonance (LSPR) sensing chip. https://www.rhino3d.com/kr/.

Regarding the main application of LSPR in developing biosensing devices, a method for the rapid detection of bacteria using the LSPR analytical system was introduced (Fig. 1). A LSPR sensing chip was fabricated by the self-assembly of AuNPs attached to a glass substrate slide, which was analyzed for the sensing signals using a spectrophotometer. The cuvette-sized LSPR sensing chip enables convenient storage of the portable detector. The LSPR sensing chip fabrication technique was simple: APTES was used as an amine group linker to attach the AuNPs to the glass substrate. Figure S2 shows that the negatively charged AuNPs were distributed uniformly in a single layer on the amine functional group-attached LSPR sensing chip surface. In the present method, the LSPR surface sensing chip was fabricated in a single step with a 12-h incubation period. In an earlier report, Yoo *et al*.^19^ fabricated the glass slide to produce an LSPR sensing surface that required several chemical reactions and complicated instruments, such as E-beam evaporator for the stepwise deposition of well-arranged Si NPs on the substrate^19^. In addition, there was no difference in the stability and reproducibility of the fabricated sensing LSPR chips. Moreover, a small amount (400 μL) of sample was sufficient to detect bacteria in food samples.

We also prepared LSPR chips using various substrates, including plastic and glass coated with gold nanoparticles. As a result, when we compared the absorbance signals of the tested LSPR chips, we observed that the absorbance of the gold nanoparticles-coated glass substrate was higher (Figure S3). In addition, after the conjugation of the aptamer to the plastic and glass substrates, the detected absorbance signals were in higher range in the case of the LSPR chip made on glass substrate (Figure S4). Thus, all further experiments were conducted using the glass substrate-based LSPR chip.

### Optimization for aptamer functionalization on the LSPR chip surface

**Figure 2:**
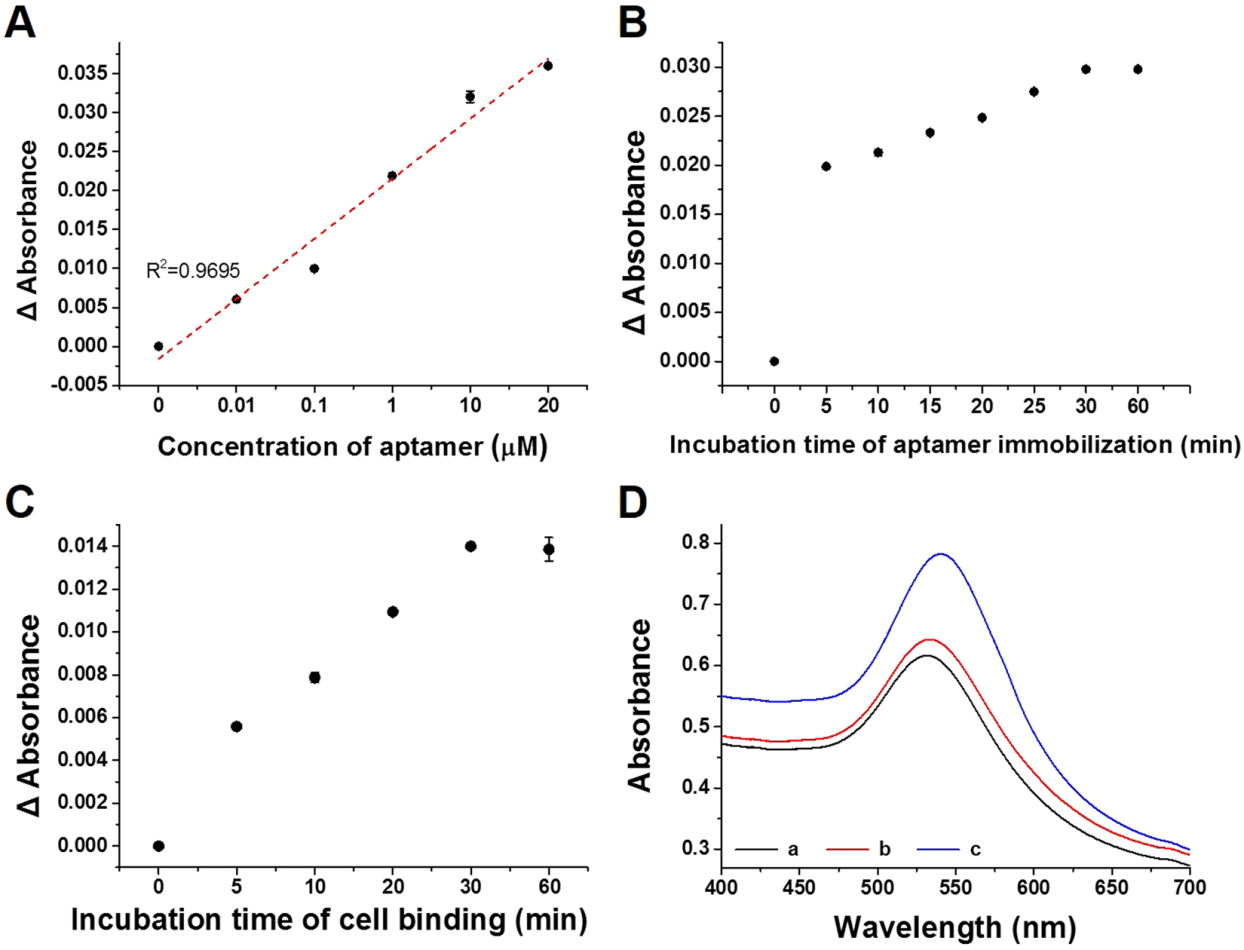
Optimum conditions for bacterial sensing using an aptamer-based localized surface plasmon resonance (LSPR) chip. (**A**) The effect of aptamer concentration on the LSPR chip; (**B**) the effect of aptamer incubation time on the LSPR chip; (**C**) the effect of bacteria binding incubation time on the LSPR chip. *S. typhimurium* (10^9^ cfu/mL) was incubated with the aptamer on the LSPR chip; (**D**) schematic representation of the detection of bacteria using the aptamer-based LSPR chip (a: bare gold chip, b: bare gold chip + aptamer, c: bare gold chip + aptamer + bacteria).

The prepared LSPR chip surface was functionalized with the *S. typhimurium* specific aptamers under optimized conditions. The synthesized aptamer is a 40-base single strand oligonucleotide with a 5-base spacer as TATGGCGGCGTCACCCGACGGGGACTTGACATTATGACAG-SH. Unlike in single-stage aptasensing detection devices, it is essential to reduce the concentration and time required for aptamer–AuNP binding because the adsorption process should be conducted on site^33^. Therefore, to optimize the minimum appropriate concentration and time required for binding the specific aptamers to the fabricated LSPR chip surface, different concentrations (0, 0.01, 0.1, 1, 10, and 20 μM) and binding incubation times (5, 10, 15, 20, 25, 30, and 60 min) were tested As a result, the LSPR peak intensity changed in response to the different concentrations of 3’ to –SH (thiol) *S. typhimurium* specific aptamer with different incubation times. Previously, thiolated aptamers or probes were observed to self-assemble readily onto the surface of the AuNPs via–Au bonding^34, 35^. Figure 2A and B show that the optimum aptamer concentration was 10 μM and the optimum binding time to the LSPR chip surface was 30 min. For the binding of the *Salmonella* cells to the aptamer, the LSPR peak intensity increased with increasing incubation time and was saturated after approximately 30 min, indicating that the maximum binding of the *Salmonella* cells to the aptamer on the sensing chip surface was achieved under these conditions (Fig. 2C). In the developed sensing system, bacterial strains were identified using the aptamer as a linker between the bacteria and AuNPs-conjugated LSPR chip. Once the AuNPs-conjugated LSPR sensing chip was functionalized with specific aptamers, the attachment of the aptamers on the LSPR chip surface must be confirmed. Therefore, the binding of aptamers with higher bacterial concentration (10^4^, 10^6^, and 10^8^ cfu/mL) was performed under the optimized conditions (10 μM aptamers and 30 min) and chip surface was further evaluated by SEM. As shown in Figure S3, the capture of lower and higher bacterial concentration of *S. typhimurium* confirmed the functionalization of the aptamers on the LSPR chip surface. The optical characteristics of the AuNPs deposited on the chip surface and the change in the LSPR intensity peak were influenced significantly by the thickness of the aptamer and bacterial interaction on its surface. The increases in the LSPR intensity peak were caused by the development of an interaction between the aptamers and bacterium on the LSPR sensing chip (Fig. 2D). Therefore, all the detection assays were performed under these optimized conditions.

### Assay procedure and sensitivity test with a pure culture of *S. typhimurium*

In the present study, the developed gold nanoparticle-aptamer-based LSPR sensing chips were tested for their detection performance against *S. typhimurium*. The ultimate aim in the development of these sensing devices is to reduce the detection cost and time with improved sensitivity; therefore, this study developed an aptamer-based sensing chip conjugated with gold nanoparticles. A previous study used antibodies conjugated with nanoparticles^36^, which were utilized for pathogen detection; however, use of aptamers has several advantages over antibodies, such as easy labeling, long-term stability, low cost, and recognition of a wide variety of targets, which suggests their potential use as a recognition probe to replace antibodies in the field of diagnostics^37^.Therefore, this study used aptamers conjugated with gold nanoparticles to improve the stability and sensitivity. In addition, the prepared sensing chips were dipped in the test solution and allowed to react for 30 min (the optimal time for bacterial binding, as determined in the previous section).

**Figure 3:**
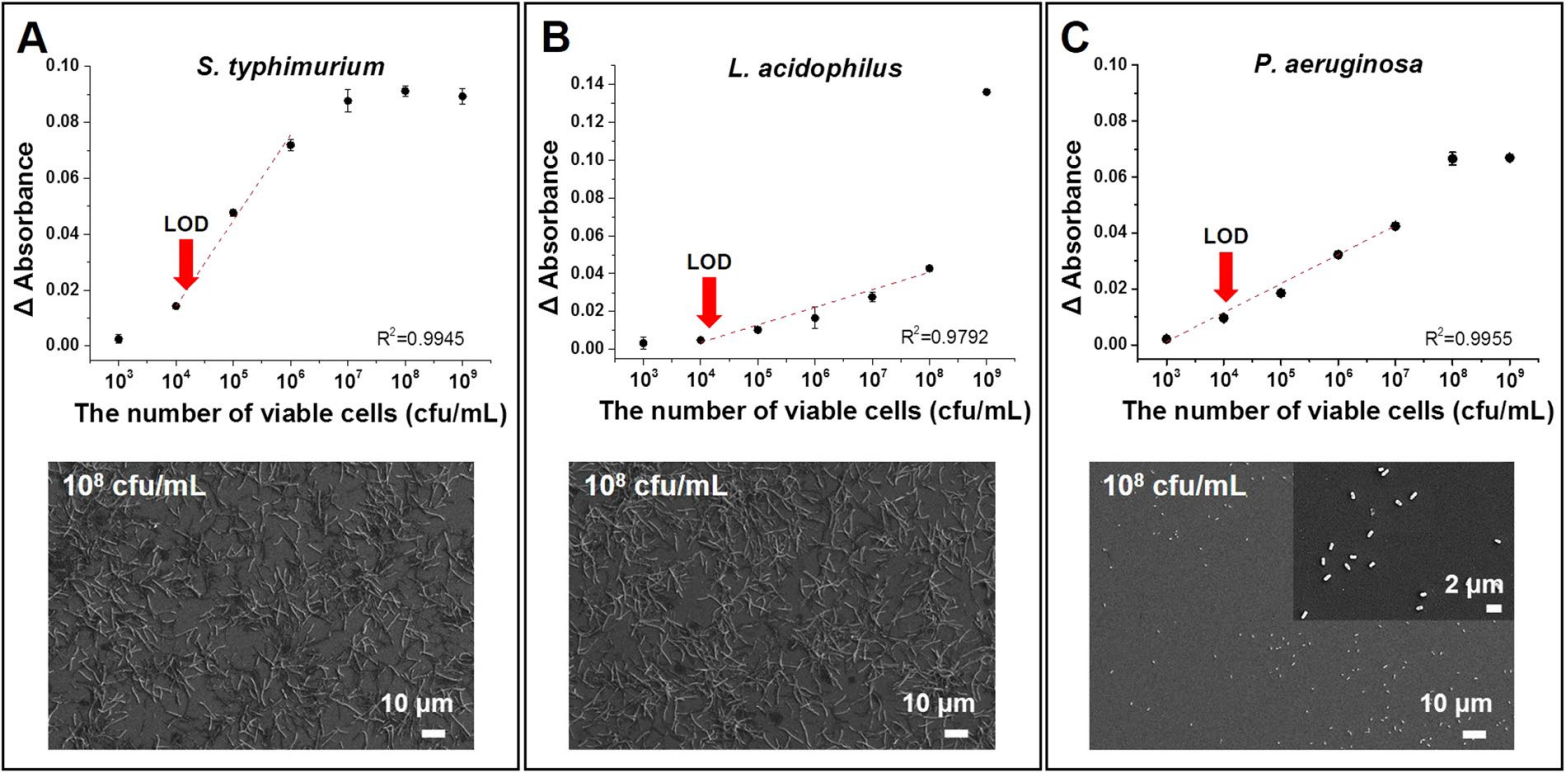
Detection of bacteria on the aptamer-based localized surface plasmon resonance (LSPR) chip. Absorbance increases from different bacterial concentrations (10^4^–10^9^ colony forming units (cfu)/mL) of (**A**) *S. typhimurium*, (**B**) *L. acidophilus*, (**C**) *P. aeruginosa*. Scanning electron microscopy (SEM) images of bound bacteria (10^8^ cfu/mL), (**A**) *S. typhimurium*, (**B**) *L. acidophilus*, and (**C**) *P. aeruginosa*.

To analyze the performance, sensitivity, and detection limit of the test assay, various concentrations of serially diluted cell cultures (10^3^, 10^4^, 10^5^, 10^6^, 10^7^, 10^8^, and 10^9^ cfu/mL) of *S. typhimurium* were used as test samples on the LSPR chip surface, followed by 30-min incubation for bacterial binding with the specific aptamers (Fig. 3A). The detection limit was calculated as the average fluorescence intensity at zero concentration with three standard deviations^31^. The LSPR peak intensity increased with increasing concentrations up to approximately 10^4^ cfu/mL, with a correlation coefficient (R^2^) of 0.9945 (Fig. 3A), suggesting good quantification capability in the linear detection range. *S. typhimurium* induced abrupt variations in the LSPR peak intensity at concentrations greater than 10^5^ cfu/mL. The SEM images also correlated with the detection pattern and confirmed that the changes in LSPR peak intensities reflected the concentrations of bacterial cells bound to the chip surface (Figure S5; Supporting information).

Previously, Yoo *et al*.^19^ reported an aptamer-based LSPR sensing method for the detection of bacterial species, and demonstrated similar results for *S*. *typhimurium* to those revealed in the present study; however, the method was modified to make it faster and simpler by reducing and modifying the pre-processing of chip preparation, such as single layering of gold nanoparticles on the glass chip. In contrast, Yoo *et al*.^19^ used repeated layering of gold and silica nanoparticles before introducing the aptamers. Cheng *et al*.^38^ also developed an aptamer-based biosensor for the detection of *S. typhimurium* using a luminescence energy transfer mechanism, and under optimal conditions, the linear detection range (R^2^ = 0.99) of *S. typhimurium* was 12 to 5 × 10^5^ cfu/mL. On the other hand, Wang *et al*.^37^ developed a quartz crystal microbalance-based aptasensor to detect *S. typhimurium*, which could detect 1.0 × 10^4^ cfu/mL of *S. typhimurium* in less than 1 h. These results confirmed that stringent efforts are still needed to develop more competitive aptasensor-based multiple detection strategies.

The method developed in this study produced the detection results within 30 to 35 min, in which the final detection using LSPR took only 5 min. The pre-preparation of the LSPR sensing chips take less than 12 h, combining 1 h for glass slide cleanup, 8–9 h for the attachment of gold nanoparticles to the glass slide, and 30 min for the conjugation of the aptamers with gold nanoparticles. The developed method can be used as a portable detection device that can be handled by untrained personnel to collect samples in detecting devices. Moreover, by modifying and reducing the fabrication steps of gold nanoparticle-aptamer-based LSPR sensing chips, the cost of the devices might be reduced without changing their detection sensitivity.

The gold nanoparticle-aptamer-based LSPR sensing chips were functionalized with other aptamers specific for *L. acidophilus and P. aeruginosa* to prove the commercial applicability of the developed detection strategy. As expected, the developed LSPR sensing chip could detect *L. acidophilus* and *P. aeruginosa* in pure cultures. The developed LSPR sensing chip showed an abrupt change in the LSPR peak intensity at a concentration of 10^7^ cfu/mL, and 10^6^ cfu/mL for *L. acidophilus* and *P. aeruginosa*, respectively (Figs 3B and 2C). SEM images also confirmed the dose dependent detection using the developed LSPR sensing chips (Figure S3). The differences in the detection limits of each bacterium might be caused by the surface properties and the growth culture conditions, and on the designed aptamers for each bacteria^39^.

In addition, in this study, we also evaluated the detection capabilities of the developed detection assay to differentiate live and heat-killed *S. typhimurium* cells (10^7^ cfu/ml). As a result, it was observed that the heat killed cells showed drastically weaker absorbance signals than live cells (Fig. S6), confirming the possibility of damaged ex-tracellular matrices of the dead cells. These findings confirm the practical application of LSPR sensing chip in the differentiation of live and dead bacterial communities.

### Cross reactivity test

Before launching a detection device for use in a rapid detection strategy, a specificity test is very important. Bacteria possess a range of analogous antigenic sites on their cell surfaces that can interfere with the specificity of a developed method^40, 41^. Therefore, it is important to determine the specificity of the developed assay for the target pathogen. The cross-reactivity of the developed assay was evaluated using *S. typhimurium* as the target pathogen, followed by other pathogenic strains, including *L. acidophilus*, *P. aeruginosa*, and *E. coli*. The results showed that no other strains showed any significant cross-reactivity (Table S2). The results showed that the LSPR absorbance peak increased significantly in response to increasing *S. typhimurium* concentrations. Specifically, there was a maximum change in LSPR peak intensity in response to 10^4^ cfu/mL relative to the negative control. Additionally, as shown in Fig. S7, the developed LSPR sensing chip did not show any significant cross-reactivity to other species of *Salmonella* including *S. tennessee* and *S. muenchen*. This emphasized that the developed detection was found species specific. Similarly, the other developed sensing chips (prepared using specific aptamers for *L. acidophilus* and *P. aeruginosa*) showed cross reactivity against the other strains, only reacting with the target strain. Few studies have confirmed that the purity of the aptamers might also affect non-specific binding^33^. Overall, these results confirmed that non-specific binding was negligible or non-existent using the developed system, suggesting that the specificity of the aptamers to the target strain was appropriate. Therefore, the LSPR sensing chip assay developed in this study could detect the target strains with high specificity.

### Applicability of LSPR sensing chip in food trials

**Figure 4:**
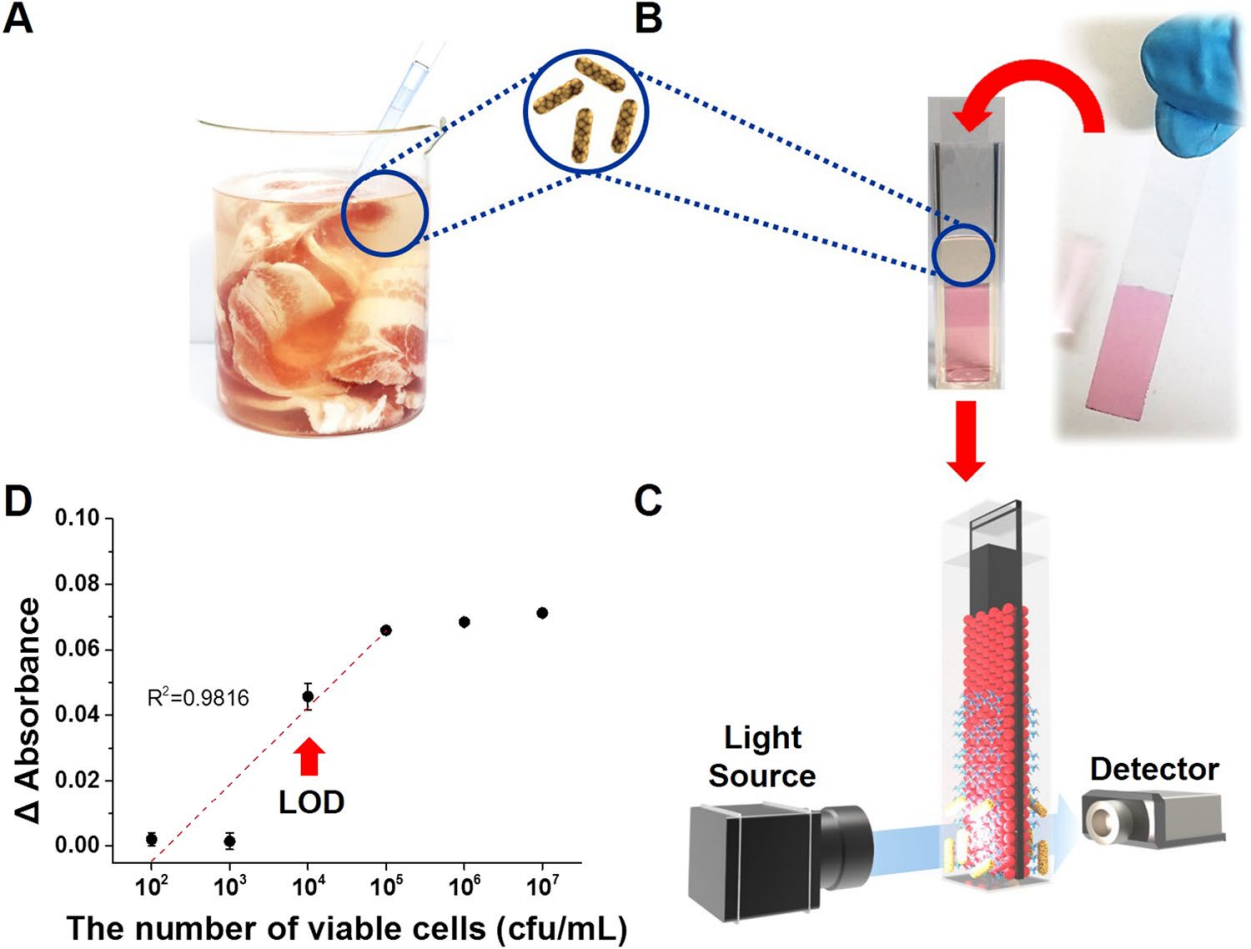
Detection of *S. typhimurium* in pork using the aptamer-based localized surface plasmon resonance (LSPR) sensing chip. (**A**) Pork extract. (**B**) Dipping the LSPR sensing chip in the pork extract. (**C**) Sensing of the binding of *S. typhimurium* to the aptamer-based LSPR sensing chip. (**D**) Determination of the concentration of *S. typhimurium* in the pork extract. https://www.rhino3d.com/kr/.

Although many of the reported applications based on the use of LSPR-based biosensors remain at a proof-of-concept level, some interesting results have appeared in fields such as food monitoring and disease diagnostics. The practical applicability of the developed biosensing LSPR chip was tested by detecting the presence of *S. typhimurium* in artificially contaminated pork meat samples (Fig. 4). The gold nanoparticle-aptamer-based LSPR sensing chip was used to measure the LSPR peak intensity in 400 µL of *S. typhimurium* artificially contaminated meat samples (Fig. 4A and B). The developed assay detected *S. typhimurium* without a pre-incubation step in artificially contaminated pork meat samples containing cells 10^4^ cfu/mL (Fig. 4C,D). The LSPR peak intensity in contaminated pork meat samples increased abruptly when the pork was contaminated with 10^4^ cfu/mL *S. typhimurium* (Fig. 4D). The detection limit in the selected food matrix was similar to that in the pure culture system (Fig. 3A), indicating that the developed method is robust and unaffected by competing molecules in the pork meat sample.

### Effect of background flora

**Figure 5:**
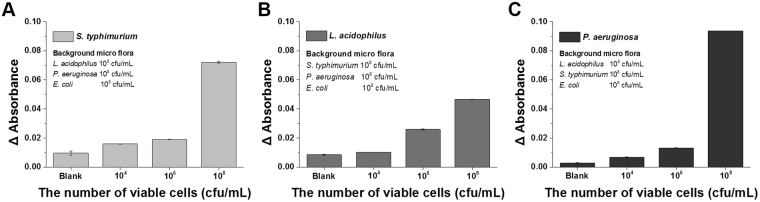
Cross reactive binding of bacteria to the aptamer-based localized surface plasmon resonance (LSPR) sensing chip. 10^8^ colony forming units (cfu)/mL of different bacteria were applied to the bar. (**A**) The concentration of *S. typhimurium* was 10^4^, 10^6^, and 10^8^ cfu/mL, while the concentrations of *L. acidophilus* and *P. aeruginosa* were fixed at 10^8^ cfu/mL. (**B**) The concentration of *L. acidophilus* was 10^4^, 10^6^, and 10^8^ cfu/mL, while the concentrations of *S. typhimurium* and *P. aeruginosa* were fixed at 10^8^ cfu/mL. (**C**) The concentration of *P. aeruginosa* was 10^4^, 10^6^, and 10^8^ cfu/mL, while the concentrations of *S. typhimurium* and *L. acidophilus* were fixed at 10^8^ cfu/mL.

The major concern for developing an applicable pathogen detection device, is that the target microorganisms must be detected in the presence of a range background microflora, whose composition will vary greatly depending on the type of test source and whose presence could cause interference during pathogen detection. Moreover, food matrices can influence the analytical process and the detection range substantially. Therefore, to analyze the effects of the background microflora, the relative detection sensitivity of the developed assay was determined using artificially contaminated pork meat samples with different concentrations of *S. typhimurium* (10^0^–10^8^ cfu/mL) and a high background concentration (10^8^ cfu/mL) of other bacterial strains, including *L. acidophilus* and *P. aeruginosa*. The background contaminant microflora had no effect on the applicability and detection sensitivity of the developed assay. As shown in Fig. 5A, even a lower level of spiked *S. typhimurium* and higher cell numbers of other background contaminants had no effect on the detection sensitivity, confirming the lack of interference from the background microflora or the food matrix. In addition, the developed method was also validated using the same strategy for LSPR sensing chips comprising aptamers specific for the other bacteria (Fig. 5B and C).

## Conclusions

A highly sensitive plasmonic-active biodevice was developed to detect a variety of pathogenic bacteria responsible for food poisoning. AuNPs (average diameter of 20 nm) were deposited uniformly on a transparent glass substrate and the developed AuNPs-based plasmonic-active sensing chips were then conjugated successfully with aptamers by a simple dipping adsorption method. The functionalized LSPR sensing chips exhibited high-sensitivity and excellent selectivity, even in the presence of different background microorganisms. The developed chip also produced a rapid (within 30–35 min) and quantitative detection of *Salmonella* with an upper detection limit of 10^4^ cfu/mL in pure culture, as well as in artificially contaminated pork meat samples (without pre-enrichment). Thus, we confirmed the practical applicability of the developed LSPR sensing chips in identifying food poisoning microorganisms in real food samples. However, the developed method has some limitations. The applicability of the developed method needs to be established in different food matrices before its commercialization. In addition, as a limitation, the device’s suitability for multiplexing should be investigated to reduce the detection processing required. Overall, this developed LSPR sensing chip has potential applications to detect hazardous pathogens in chemical and clinical laboratory settings, the agricultural industry, and the food industry, and for environmental monitoring.

### Data availability

The authors declare that all the other data supporting the finding of this study are available within the article and from the corresponding author on reasonable request.

## Electronic supplementary material

Supplementary Information
