# Supplementary material for: Development of gold nanoparticle-aptamer-based LSPR sensing chips for the rapid detection of Salmonella typhimurium in pork meat
Source: Sci Rep. 2017 Aug 31;7:10130. doi: 10.1038/s41598-017-10188-2 (PMC5579046; doi:10.1038/s41598-017-10188-2)
Supplement: Supplementary file 1 — Supplementary Information [file 41598_2017_10188_MOESM1_ESM.doc]

**Supplementary Dataset 1**

**Development of gold nanoparticle-aptamer-based LSPR sensing chips for the rapid detection of *Salmonella typhimurium* in pork meat**

Seo Yeong Oh^1,+^, Nam Su Heo^1,+^, Shruti Shukla^2^, Hye-Jin Cho^3^, A.T. Ezhil Vilian^2^, Jinwoon Kim^1^, Sang Yup Lee^4^, Young-Kyu Han^2^, Seung Min Yoo^4^,* & Yun Suk Huh^1^,*

^1^ Departemnt of Chemical Engineering, Inha University, 100 Inha-ro, Nam-gu, Incheon 22212, Republic of Korea.

^2^ Department of Energy and Materials Engineering, Dongguk University-Seoul, 30 Pildong-ro 1-gil, Seoul 04620, Republic of Korea.

^3^ Reliability Assessment Center for Chemical Materials, Korea Research Institute of Chemical Technology (KRICT), 141 Gajeong-ro, Yuseong-gu, Daejeon 34114, Republic of Korea.

^4^ Department of Chemical and Biomolecular Engineering (BK21 plus program), KAIST, Daejeon, 34141, Republic of Korea

**^+^These authors contributed equally to this work**

***Corresponding authors:**

E-mail: smyoo@kaist.ac.kr (Dr. S.M. Yoo)

E-mail: yunsuk.huh@inha.ac.kr (Prof. Y.S. Huh)

**Table S1. The sequences of the bacterial species-specific aptamers used in this study (CH_2_)_3_-SH at 3’**

| **Species** | **Sequence (5’ to 3’)** | **Source** |
| --- | --- | --- |
| *Lactobacillus acidophilus* | AGCAGCACAGAGGTCAGATGTAGCCCTTCAACATAGTAATATCTCTGCATTCTGTGTGCCTATGCGTGCTACCGTGAA | KCTC 3164 |
| *Salmonella typhimurium* | TATGGCGGCGTCACCCGACGGGGACTTGACATTATGACAG | KCTC 2421 |
| *Pseudomonas aeruginosa* | CCCCCGTTGCTTTCGCTTTTCCTTTCGCTTTTGTTCGTTTCGTCCCTGCTTCCTTTCTTG | ATCC 15692 |

**Table S2.** Cross-reactivity (specificity) of the developed *S. typhimurium* LSPR sensing chip against other genus bacterial strains.

| **Species** | **LSPR sensing** |
| --- | --- |
| *Pseudomonas aeruginosa* | - |
| *Lactobacillus acidophilus* | - |
| *Escherichia coli* | - |
| *Salmonella typhimurium* | + |

**
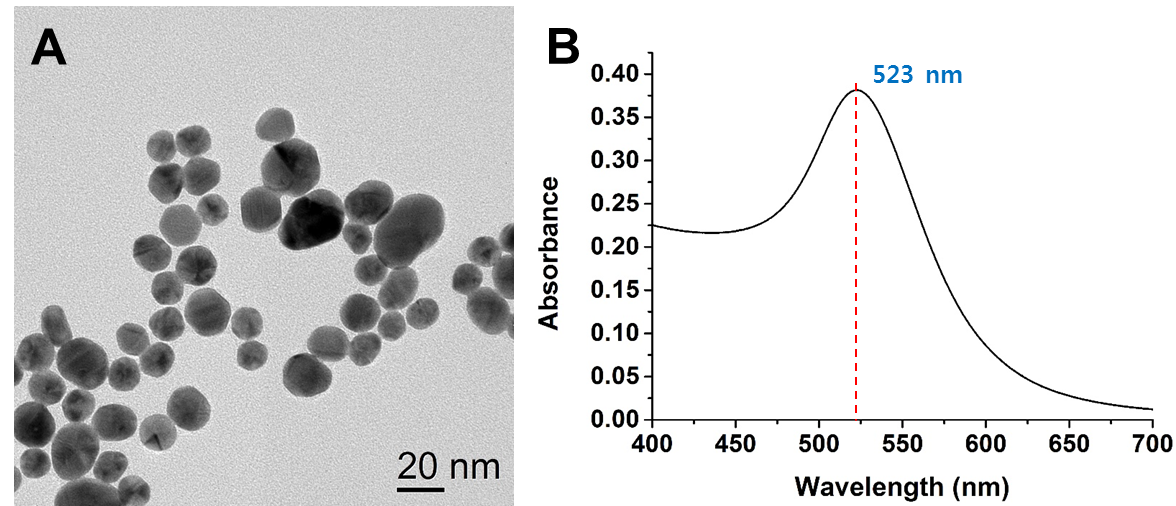
**

**Figure S1.** (A) TEM image and (B) UV spectra of synthesized AuNPs.


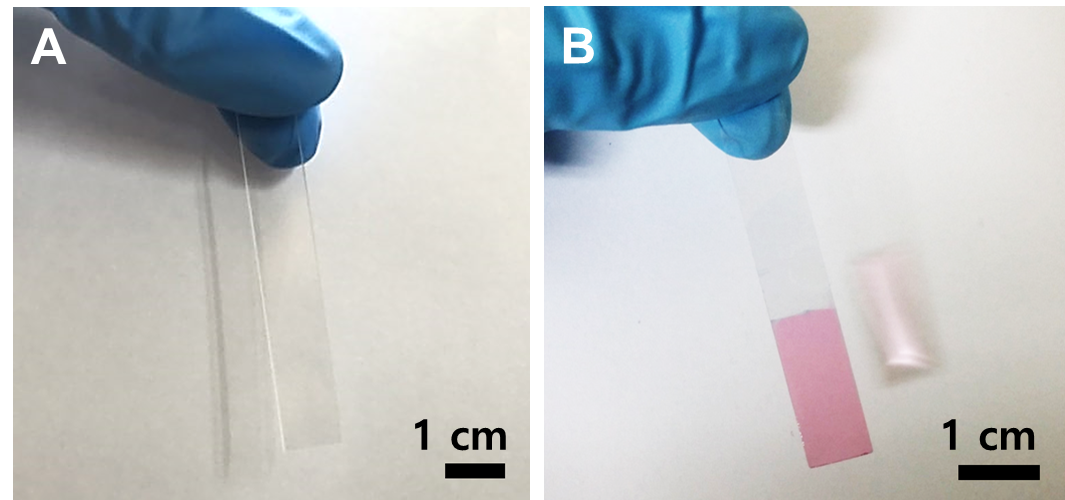


**Figure S2.** (A) glass slide without AuNPs fabrication(B) glass slide with AuNPs fabrication.


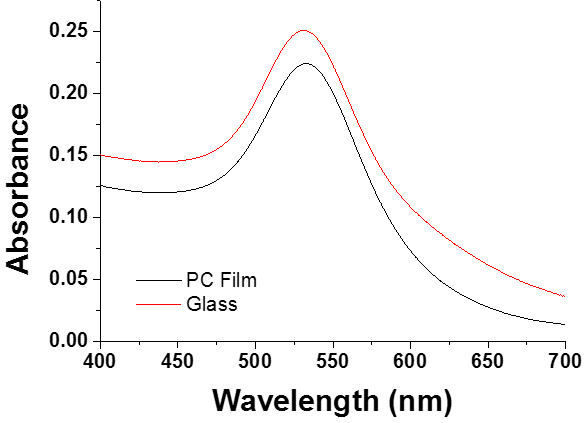


Figure S3. Absorbance of plastic and glass substrate.


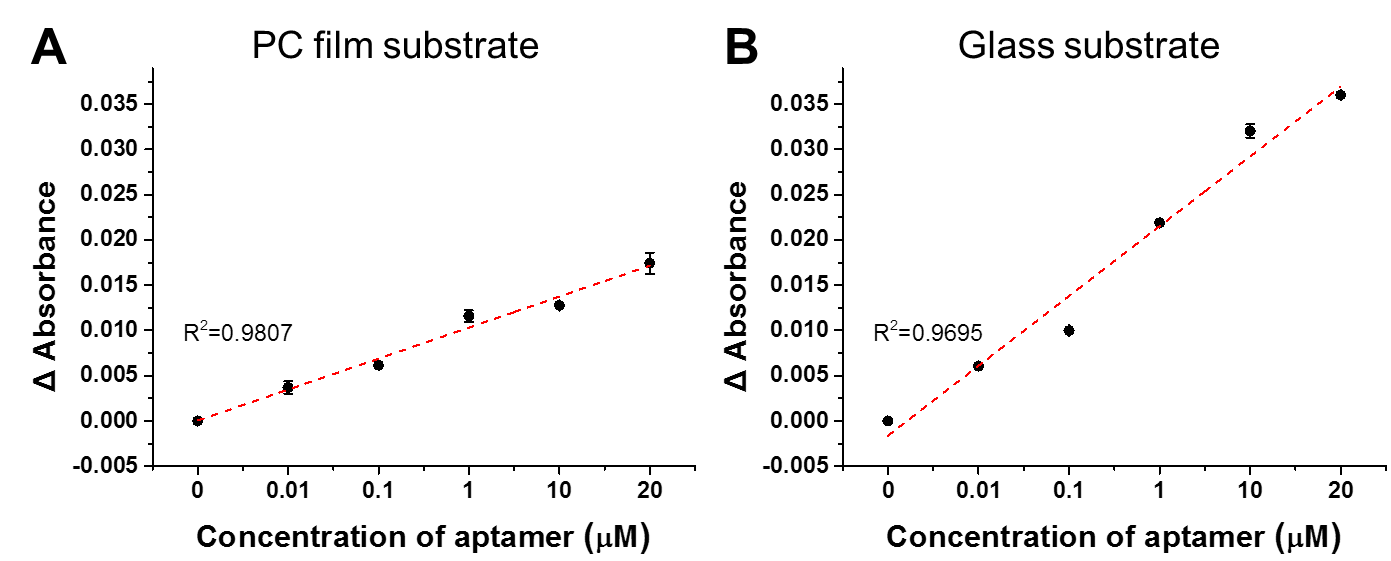


**Figure S4.** Effect of aptamer concentrations on localized surface plasmon resonance (LSPR) chip. (A) LPSR chip of PC film substrate, (B) LSPR chip of glass substrate.


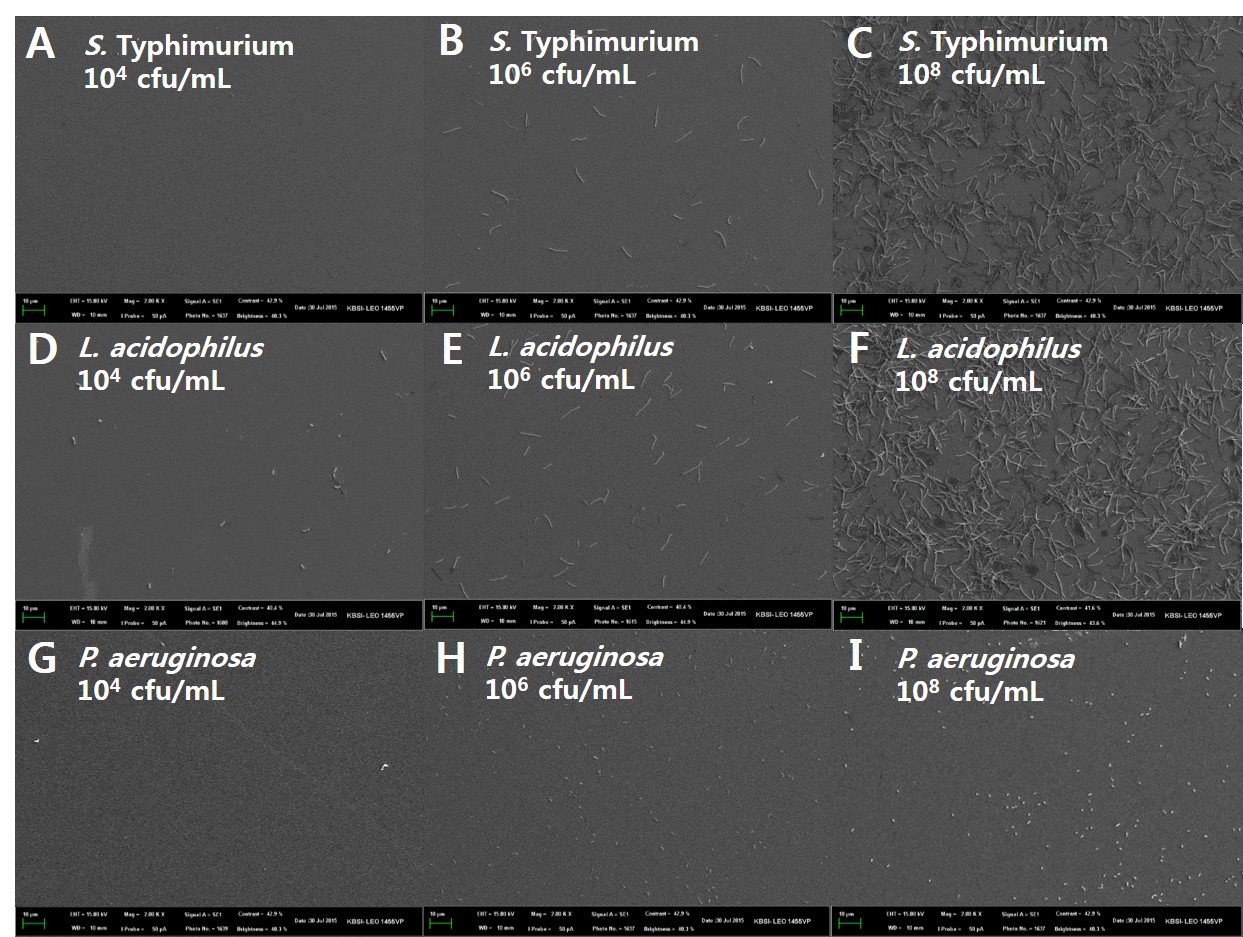


**Figure S5.** SEM images of bound bacteria (different concentrations) on the aptamer functionalized localized surface plasmon resonance (LSPR) chip (A), (B), (C) *S. typhimurium,* (D), (E), (F) *L. acidophilus* and (G), (H), (I) *P. aeruginosa*.

**Figure S6. Detection of live and heat-killed *S. typhimurium* cells using the developed detection assay.** The data represent the mean±S.D. of three measurements.


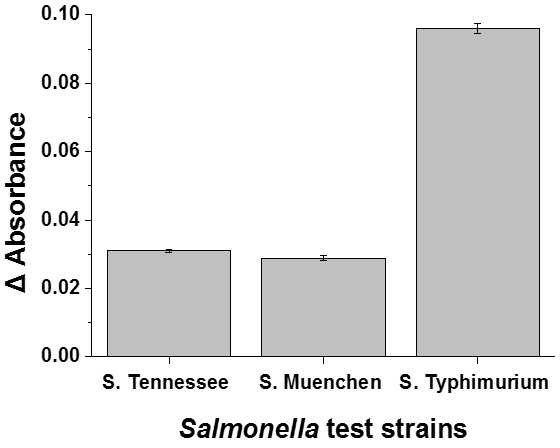


**Figure S7. Cross reactivity of developed localized surface plasmon resonance (LSPR) sensing chip against other *Salmonella* strains .** The data represent the mean±S.D. of three measurements.
